# Inhibitory processes in toddlers: a latent-variable approach

**DOI:** 10.3389/fpsyg.2014.00381

**Published:** 2014-04-30

**Authors:** Elena Gandolfi, Paola Viterbori, Laura Traverso, M. Carmen Usai

**Affiliations:** Department of Educational Science, University of GenoaGenoa, Italy

**Keywords:** inhibitory processes, executive functions, latent structure, early childhood, confirmatory factor analysis

## Abstract

The aim of this study was to investigate the nature of inhibitory processes in early childhood. A confirmatory factor analysis was used to examine the latent structure of inhibitory processes in day-care center children aged 24–32 months and in preschool children aged 36–48 months. The best fit to the data for the younger sample was a single undifferentiated inhibition factor model; in older children, a two-factor model was differently identified in which response inhibition and interference suppression were distinguished.

## Introduction

Inhibitory processes are considered important components of cognition that affect an individual's ability to function in everyday life. Using a broad definition, inhibitory processes refer to the ability to control one's mental processes and responses, to ignore an internal or an external prompt, and to perform an alternative action (Diamond, 2013).

During childhood, inhibitory processes have been found to affect different aspects of child functioning, such as self-regulatory behaviors (e.g., Rueda et al., 2005), theory of mind (e.g., Carlson et al., 2002), and the internalization of conduct standards (e.g., Kochanska et al., 1996). Inefficient inhibitory processes have been linked to several developmental disorders, such as attention deficit hyperactivity disorder (Barkley, 1997; Ozonoff and Jensen, 1999; Schachar et al., 2000), obsessive compulsive disorder, and autistic spectrum disorder (Ozonoff et al., 1991; Robinson et al., 2009).

In the recent literature, inhibitory control has been considered one aspect of the multi-component construct of executive function that proves to be clearly separate from other executive dimensions, such as shifting and working memory (WM), in adults (Miyake et al., 2000) and older children (Lehto et al., 2003). However, in younger children, the separability of different executive functions remains a matter of debate.

Using a confirmatory statistical approach, previous studies have found that a single undifferentiated executive control factor was the most appropriate means of describing the executive latent structure in early childhood and in preschoolers (Wiebe et al., 2008, 2011; Hughes et al., 2010; Willoughby et al., 2010; Fuhs and Day, 2011). Diverging from previous results, Miller et al. (2012) reported that a two-factor model, which consisted of WM and inhibition, fitted the data better in a sample of preschoolers between the ages of 3 and 5 years than did a single-factor model or a three-factor model composed of WM, inhibition, and shifting. Similarly, Usai et al. (2014) found that a two-factor model provided the best fit for the data, with inhibition as a separate dimension from a working memory-flexibility factor, at both 5 and 6 years of age. These studies suggest that an emerging differentiation of EF processes is already apparent in early childhood and that inhibitory processes emerge as a separate dimension as early as the preschool years.

Although a fairly large body of literature exists on inhibitory processes and their role in child functioning and development, a precise definition of inhibition remains elusive.

### Inhibition: single or multiple processes?

An important shift in the research on inhibition concerns the idea that inhibition may be better conceptualized as a set of functions than as a unitary construct (Dempster, 1992; Nigg, 2000). Of course, this approach implies that there are commonalities as well as differences between the various inhibitory functions.

In his review, Nigg (2000) distinguished between the effortful inhibition of a motor or cognitive response and the automatic inhibition of attention. He included in the first category four different types of inhibition: (a) interference control, which is the ability to prevent interference due to resource or stimulus competition; (b) cognitive inhibition, which involves suppressing non-pertinent thoughts to preserve other processes, such as WM or attention; (c) behavioral inhibition, which refers to the ability to overcome a prepotent response or a prompted but socially inappropriate response; and (d) oculomotor inhibition, which involves suppressing a reflexive saccade.

The taxonomy of Nigg (2000) can be considered a theoretical attempt to describe the different inhibitory functions. Friedman and Miyake (2004), using a latent variable analysis, subsequently distinguished between three main forms of inhibition (prepotent response inhibition, resistance to distractor interference, and resistance to proactive interference), more or less in accordance with the taxonomy of Nigg (2000).

Prepotent response inhibition was defined as the ability to intentionally prevent a dominant, automatic, or prepotent response; resistance to distractor interference was identified as the ability to overcome an interference that is external to the individual and irrelevant to the current task; and resistance to proactive interference was defined as the ability to control interference from previous tasks. In adults, the results suggested that the term “inhibition” could not be overextended to different processes. A common inhibition ability was found, which was represented by inhibition of action (prepotent response inhibition) and inhibition of attention (resistance to distractor interference), both of which involve the ability to actively maintain critical goal-related information. Surprisingly, resistance to proactive interference was unrelated to both the prepotent response inhibition and resistance to distractor interference, which suggests that this type of cognitive inhibition acts as an independent dimension.

More recently, in a review of the literature, Diamond (2013) suggested that inhibitory control could be divided into three main components: inhibition at the level of thought and memories (cognitive inhibition), inhibition at the level of attention (executive attention), and inhibition at the level of behavior (response inhibition). Cognitive inhibition and executive attention are the mechanisms underlying interference control, which involves both the ability to suppress interfering (or prepotent) mental representations and the ability to ignore (or inhibit attention to) particular stimuli to attend to other stimuli based on one's goals or intentions.

In contrast, the response inhibition component involves the ability to regulate one's behavior and control one's emotions to support the regulation of a behavior. This ability involves preventing impulsive behaviors when completing a task despite being faced with distractions or other competing stimuli. In children, this behavioral self-control is facilitated if there is sufficient time between the triggering stimulus and the response the child should produce (Simpson et al., 2012).

A distinction between the capacity to suppress prepotent but inappropriate responses (response inhibition) and the ability to filter out irrelevant information in the environment (interference monitoring and suppression) was also suggested by Bunge et al. (2002), who found differences in the regions of neural activation associated with response inhibition and interference suppression. This distinction is based on the differences between tasks that constitute potentially conflicting dimensions, such as the *Flanker* tasks, and univalent tasks, in which only a single feature is presented and the conflict is between two response options to the same stimulus. This situation creates a conflict between the habitual response and a less familiar arbitrary response, as in the *Day/Night Stroop* task. It has been suggested (Blasi et al., 2006) that in tasks requiring interference suppression, both the response conflict and the process of filtering out incongruent information within the stimulus are present.

### Inhibitory control development

The presence of inhibitory processes in toddlerhood and preschool-aged children has been established in many studies (Kochanska et al., 1997; Diamond, 2002; Jones et al., 2003; Carlson, 2005; Garon et al., 2008). However, given that the term inhibition has been variously used in the literature, as noted previously, it is not easy to extract a general trajectory of the development of inhibitory processes from the literature.

In their review, Best and Miller (2010) suggested that significant development of inhibitory processes occurs in the preschool years. By age 4, children show signs of successful performance on both response inhibition tasks and complex inhibition tasks, which require substantial WM. Inhibition continues to improve, especially from 5 to 8 years of age and particularly for tasks that combine inhibition and WM (Gerstadt et al., 1994; Carlson, 2005). According to Best and Miller (2010), these later improvements are unlikely to reflect fundamental cognitive changes, such as a preschooler's acquisition of the rule-formation ability, which is necessary for performing tasks such as the *Dimensional Change Card Sort*. Instead, the fundamental changes in cognition consist of quantitative improvements in accuracy.

Rueda et al. (2005), distinguishing response inhibition and inhibition in the attentional domain from conflict resolution, claimed that the ability to resolve conflict is the most important milestone in EF development, which develops slowly in the first 2 years of life and improves noticeably between 2 and 5 years of age. Similarly, Clark et al. (2013), using growth curve modeling to describe the growth trajectories of inhibitory control and cognitive flexibility, found a sizeable increase in these abilities, particularly during the period of 3–4 years of age. The authors suggested that this accelerated growth may reflect a qualitative change in executive processing. They also found differences in the developmental trajectories of different task conditions related to different cognitive demands.

Garon et al. (2008) distinguished between simple and complex inhibition processes, referring particularly to tasks that are employed to explore the inhibitory processes during early childhood, and classified them according to WM demands. These authors ascribed paradigms, such as the *Don't*, the *Delay gratification*, the *Object retrieval*, and the *Antisaccade*, to simple inhibition tasks. Conversely, they included the *Simon-like* tasks, the *Flanker* tasks, the *Less is more* task, the *Hand game* task, and the *Knock and tap* task as examples of complex inhibition paradigms because these tasks require the resolution of conflict between dominant and subdominant responses and, consequently, involve greater levels of top-down control. Best and Miller (2010) also included the *Dimensional Change Card Sort* in these complex tasks because it determines a prepotent response during the pre-switch phase that must later be inhibited. In the post-switch phase, the child is asked to sort the same cards by the other dimension that conflicts with the previous one, which remains visible.

### The present study

The aim of the present study was to investigate the latent organization of inhibitory processes in early childhood. Following the hypothesis of Bunge et al. (2002), we considered two dimensions of inhibition: *response inhibition* with low WM demands and *interference suppression*, which is associated with higher WM demands and requires the individual to address interference or conflict from recently presented information or to filter out incongruent information. We performed a cross-sectional study in which a confirmatory factor analysis (CFA) was used to investigate the latent structure of inhibitory processes in children aged 24–32 and 36–48 months. Research investigating the underlying construct of inhibition at these age levels is currently absent from the literature.

Two different models of inhibition were tested. First, we considered a unitary factor model based on earlier studies indicating that a single, undifferentiated, executive control factor was the most appropriate for describing the executive latent structure in preschoolers (Wiebe et al., 2008, 2011; Hughes et al., 2010). We subsequently examined a two-factor model in which *response inhibition* was distinguished from *interference suppression*. To test these two models, we chose measures that assessed the ability to suppress prepotent but inappropriate responses (*response inhibition*) and the ability to manage the interference of potentially conflicting features of the task (*interference suppression*), as suggested by Bunge et al. (2002).

In *response inhibition* tasks, the conflict is between two response options to the same stimulus, namely, the habitual response and a less familiar response. For example, the *Circle Drawing Task* and the *Tower Building* task require the ability to suppress an impulsive motor response when a task calls for it; similarly, in the *Bear/Dragon* task, the child needs to selectively suppress commanded actions in response to a stimulus based on a rule. In the *Day/Night Stroop* task, the child must suppress the tendency to produce a dominant response (say “day” when a card with a sun is presented) in favor of a subdominant response (say “night” when a card with a sun is presented). These tasks are examples of univalent displays in which only a single feature is presented and the conflict is between two response options to the same stimulus feature (Martin-Rhee and Bialystok, 2008).

*Interference suppression* tasks require the child to select a piece of information from a complex stimulus that is misleading and in which interfering features of the stimulus must be inhibited. These latter tasks involve greater levels of cognitive control, are associated with higher WM demands, and require individuals to filter out irrelevant information.

For example, in the *Fish task*, the child must respond to a central target flanked by distractors whose interference must be inhibited. In the *Reverse Categorization* task and the *Dimensional Change Card Sort* task, children must classify objects or cards by considering their different features, inhibiting the sorting rule previously learned. In particular, children must inhibit their attention to a dimension of the stimulus that was previously useful to solve the task and attend to a different aspect of the same stimulus. The *Animal House* task requires the child to match animal stickers with a color following a precise association rule and inhibiting the previous animal-color association each time.

To our knowledge, there has been no systematic attempt to empirically evaluate these dimensions of inhibition in early childhood.

## Materials and methods

### Participants

The present study involved two samples of 130 typically developing children: 60 children between the ages of 24 and 32 months (mean age = 28.41 months; *SD* = 2.68; *n* = 25 males and 35 females) in their last year of day-care and 70 children between the ages of 36 and 48 months (mean age = 42.35 months; *SD* = 3.18; *n* = 34 males and 36 females) in their first year of preschool. The participants were recruited by contacting six day-care centers and four kindergartens in the largest town in a northern region of Italy. Written parental informed consent was obtained before the participating children were admitted to the assessment sessions. Parents also completed a socioeconomic and educational background questionnaire: the mother's education level ranged from 8 to 18 years (mean = 13.7 years), and the father's education level ranged from 5 to 18 years (mean = 11.57 years); the mother's annual income ranged from 0 to 42,000 € (mean = 17,000 €), and the father's annual income ranged from 14,000 € to 42,000 € (mean = 22,000 €). Children with documented health problems, such as neurological, psychiatric or developmental disorders, or whose primary language spoken at home was not Italian were excluded from the study.

### Procedure

The children were tested individually in a quiet room of their day-care center or preschool during a 30- to 40-min session. Researchers and trained graduate students administered and scored all tests. A battery of inhibitory tasks, varying in format and response demands, were administered to the children in a standard order.

### Inhibitory measures

A battery of tasks was employed to assess two inhibitory abilities: *response inhibition*, which is the ability to suppress a prepotent but inappropriate response to a stimulus, and *interference suppression*, which is the ability to address the interference of potentially conflicting characteristics of a stimulus.

The following measures were administered to children aged 24–32 months:

1. Response inhibition:
  The *Circle Drawing Task* (Bachorowski and Newman, 1985) assesses the ability to control an ongoing motor response. A circle is drawn on a cardboard square. The circle has a small arrow printed above its line to indicate the starting point and the direction of the tracing. The task is administered under two conditions: first with neutral instruction (“*Trace the circle with your finger*”), followed by an inhibition instruction (“*Trace the circle again, but this time as slowly as you can*”). The score is calculated as the proportion of the slowdown to the total time using the following formula: T1−T2/T1+T2, where T1 and T2 are the times recoded for the first and the second trials, respectively.
  The *Tower Building* (Kochanska et al., 1996) evaluates the ability to take turns and to inhibit a prepotent response as in a go-no go task. The children are asked to take turns with the experimenter to build a tower using 20 wooden blocks (10 red and 10 blue). The score indicates the number of correct turns (range: 0–10).
2. Interference suppression:
  The *Fish Task* (Viterbori et al., 2012; adapted from Rueda et al., 2004) evaluates visual interference using an adaptation of the flanker paradigm (Eriksen and Eriksen, 1974). This is a forced-choice task in which children are required to point at where a centrally located target fish is oriented, despite the presence of interfering stimuli (other fishes) whose interference must be inhibited. There are 14 trials: 2 training trials, 6 congruent trials with the target and the interfering stimuli oriented in the same direction and 6 incongruent trials with the target and the interfering stimuli oriented in the opposite direction. Congruent and incongruent trials are randomly presented. The accuracy in the incongruent trials is scored (range: 0–6).
  The *Animal House* (adapted from WPPSI; Wechsler, 1973) measures a child's ability to choose the correct association between stimuli (i.e., animal-color) by filtering out the other competing possibilities. The examiner shows the child three stickers, which represent a duck, a mouse and a frog, that are each matched with a colored house: the duck is matched with a red house, the mouse is matched with a blue house and the frog is matched with a yellow house. The child is then asked to correctly match 20 animal stickers (duck, mouse, and frog) with 20 different colored houses (red, blue, and yellow). In order to reduce WM load, before starting, the experimenter provided the child with an example of the matching rules which remained visible during the whole task. The score is obtained by calculating the total number of correctly matched stickers (range: 0–20).
  The *Reverse Categorization* (Carlson et al., 2004) evaluates the ability to classify an object according to different rules. The task requires an individual to resolve a conflict generated by the previous presentation of a classification rule, which subsequently represents a source of interference. Children are introduced to two buckets and 12 blocks (six small and six big). The experimenter, using demonstration and verbal explanation, asks the child to sort big blocks into the “big” bucket and little blocks into the “little” bucket (pre-switch phase). Then, the experimenter reverses this categorization scheme (post switch phase) and suggests playing a “silly game” in which the children have to sort big blocks into the “small” bucket and small blocks into the “big” bucket. For each trial, the experimenter repeats the rule and then identifies the current block as big or small. There are 12 test trials for each phase, and no feedback is given. The score is the number of correct classifications in the post-switch phase (range: 0–12).

The following measures were administered to children aged 36–48 months:

1. Response inhibition:
  The *Circle Drawing Task* (Bachorowski and Newman, 1985) is the same as described above.
  The *Bear/Dragon* (Reed et al., 1984) assesses the ability to inhibit or activate a motor response following a rule, in a similar way as in a *go no-go* task. The experimenter introduces children to a “nice” bear puppet and a “naughty” dragon puppet. The children are told that in this game, they are to do what the bear asks them to do (e.g., “touch your nose”), but not to do what the dragon asks. After practicing, there are 10 test trials with the bear and dragon commands in alternating order. The children are seated at a table throughout the task, and all actions involve hand movements. The performances on the bear and dragon trials are considered to be an index of self-control. The tasks are scored as follows: “0 indicates a movement or response when the dragon asks and no movement when the bear asks; 3 indicates no movement when the dragon asks and a movement or response when the bear asks” Also partial credits were scored: 2 indicates a partial movement or response when the bear asks, and a wrong movement when the dragon asks; 1 indicates a wrong movement when the bear asks and a partial movement or response when the dragon asks. The score ranges from 0 to 30.
  The *Day/Night Stroop* (Gerstadt et al., 1994) assesses the ability to inhibit a prepotent verbal response and to activate an alternative verbal response. The experimenter presents a white card with a yellow sun and a black card with a white moon and stars on it. The children are instructed that in this game, they must say “Night” for the sun cards and “Day” for the moon cards. There are 16 test trials with each card presented in a fixed and pseudorandom order. There are no breaks or rule reminders. The accuracy (the number of correct items out of 16) is recorded (range: 0–16).
2. Interference suppression:
  The *Dimensional Change Card Sort* (DCCS, Zelazo, 2006) evaluates the extent to which young children between three and six years of age are able to remember two sets of rules, apply them and then switch the rules. This task requires children to address the interference generated by the previous sorting rule. Children are introduced to two recipe boxes, which have rectangular slots cut in the top. Target cards (a red rabbit and a blue boat) are affixed to the front of the boxes. The experimenter presents a series of cards (red and blue rabbits and boats) and instructs the children to place all the rabbits in the box with the red rabbit and all the boats in the box with the blue boat in the “shape game.” After five consecutively correct trials, the experimenter asks the children to stop playing the “shape game” and to play the “color game” (post-switch phase). In this case, all the red items must go in the box with the red rabbit affixed, and all the blue items must go in the box with the blue boat affixed. In the third sorting phase (border phase), the experimenter explains that if there is a black border on a card, then the children must sort according to color; however, if there is no border, then they must sort according to shape. There are 24 trials (6 for the pre-switch phase, 6 for the post-switch phase, and 12 for the border phase); the score represents the number of correct responses (0–24).
  The Fish Task (Viterbori et al., 2012; adapted from Rueda et al., 2004) and the Animal House task (adapted from WPPSI; Wechsler, 1973) are the same as described above.

## Results

### Descriptive statistics

The descriptive statistics for all inhibitory measures are shown in Table 1.

**Table 1:** **Descriptive statistics for the inhibitory measures used in 24–32 month sample and in 36–48 month sample**.

	**24–32 month sample**						**36–48 month sample**				
	***N***	**Mean**	***SD***	**Skewness**	**Kurtosis**		***N***	**Mean**	***SD***	**Skewness**	**Kurtosis**
**RESPONSE INHIBITION**											
Circle	56	0.18	0.23	−0.07	0.51	Circle	70	0.33	0.24	−0.09	−0.56
Tower	56	5.35	2.8	−0.11	−0.73	Bear	69	19.79	6.62	0.41	−1.05
–	–	–	–	–	–	D/N	70	11.28	4.8	−1.21	0.49
**INTERFERENCE SUPPRESSION**											
Fish	57	2.61	1.67	−0.05	−0.79	Fish	70	4.45	1.85	−0.91	−0.49
Animal	60	9.47	3.75	0.77	1.12	Animal	70	18.00	3.33	1.98	3.13
Reverse	60	9.65	2.74	−0.75	0.9	DCCS	70	17.27	4.22	−2.09	4.87

*Circle, circle drawing task; Tower, tower building; Fish, fish task; Animal, animal house; Reverse, reverse categorization; Bear, bear and dragon; DCCS, dimensional change card sort; D/N, day/night stroop*.

No outliers were identified. The missing values for all measures ranged from 0 to 3%.

All dependent variables displayed adequate distributional characteristics, without substantial skewness or kurtosis. For both age range measures, skewness and kurtosis coefficients were relatively low, except for the *Animal House* task (for the 36–48 month sample), for which raw scores were transformed using an arcsine transformation, and the *DCCS* task, for which raw scores were transformed using a logarithmic transformation [Log10 (max range + 1 − x)]. The transformed descriptive statistics for the *Animal House* task and the *DCCS* task were as follows: *Animal House*: mean = 1.27, *SD* = 0.38, skewness = −1.06, kurtosis = 0.06; *DCCS*: mean = 0.85, *SD* = 0.21, skewness = 0.60, kurtosis = 0.99.

The mean scores obtained by both samples in the common inhibition measures were compared using an independent samples *t*-test. The results showed significantly better task performance for the older children in all tasks, including the *Circle Drawing Task* [*t*_(124)_ = −3.58, *p* < 0.001], the *Fish Task* [*t*_(125)_ = −5.81, *p* < 0.001] and the *Animal House* task [*t*_(127)_ = −13.66, *p* < 0.001].

### Correlations

Zero-order (Pearson) and partial correlations controlled for age (upper triangle, Table 2) among inhibitory measures were performed.

**Table 2:**
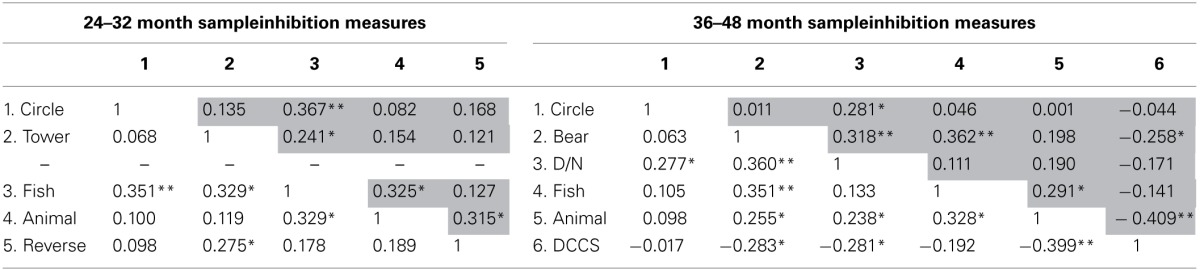
**Zero order and partial correlation controlled for age (upper triangle) between inhibitory measures in 24–32 month sample and in 36–48 month sample**.

Circle, circle drawing task; Tower, tower building; Fish, fish task; Animal, animal house; Reverse, reverse categorization; Bear, bear and dragon; DCCS, dimensional change card sort; D/N, day/night stroop. ^*^p < 0.05; ^**^p < 0.01. The negative values of the DCCS are due to the mathematical transformation used.

Consistent with the findings of previous studies (Wiebe et al., 2011), the correlations were generally low in both samples.

In the 24–32 month sample, the *response inhibition* tasks were positively correlated with the *Fish Task*, which was considered to be an *interference suppression* task. In particular, the response to the incongruent condition of the *Fish Task* showed a significant correlation pattern with the slowdown motor response of the *Circle Drawing Task* and with the number of correct turns in the *Tower Building* task; these associations remained significant after controlling for age. Moreover, the *Tower Building* task showed a positive correlation with the *Reverse Categorization* task.

Among the *interference suppression* tasks, the number of correct items on the *Animal House* task correlated moderately with the correct responses on the *Fish Task* and with the number of correct items in the post-switch phase of the *Reverse Categorization* task. In this last case, the association was significant only after controlling for age.

In the 36–48 month sample, the *response inhibition* tasks correlated positively with one another. In particular, the ability to inhibit the interference to activate an alternative response of the *Day/Night Stroop* task was significantly correlated with the *Circle Drawing Task* and the ability to inhibit a prepotent response in the *Bear/Dragon* task. All of these tasks share the ability to inhibit an impulsive or a dominant response.

The *interference suppression* tasks were significantly correlated with one another. The *Animal House* task, which evaluates the ability to resolve a conflict generated by the previous presentation of a different classification rule, was positively correlated with the ability to manage interfering stimuli, as evaluated by the *Fish Task*, and with the ability to suppress the non-pertinent learned rule in a misleading situation of the *DCCS* task.

The *interference suppression* tasks were also correlated with *response inhibition* measures, such as the *Bear/Dragon* task and, in the case of the *Animal House* task and the *DCCS* task, with the *Day/Night Stroop* task.

### Confirmatory factor analysis

To identify which model would be more useful to explain the observed data, a series of CFAs, based on covariance matrices, were performed using EQS 6.1 software^1^ (Bentler, 2006). Multiple fit indices were considered for comparing models (for an example of an extensive description, see Schermelleh-Engel et al., 2003): the *X*^2^ statistic, the root mean square error of approximation (RMSEA), the standardized root mean squared residual (SRMR), the Bentler's Comparative Fit Index (CFI), the Non-Normed Fit Index (NNFI) and the Akaike Information Criterion (AIC).

The *X*^2^ test was used to evaluate the appropriateness of the CFA model: non-significant *X*^2^ values indicated a minor difference between the covariance matrix generated by the model and the observed matrix and, thus, an acceptable fit. The RMSEA and the SRMR are the absolute fit indices, which assess how well an a priori model reproduces the sample data (Hu and Bentler, 1999). The RMSEA, which is a measure of the approximate fit in the population, measures how closely the covariances predicted by the model match the actual covariances. RMSEA values = 0.05 represent a good fit, values between 0.05 and 0.08 represent an adequate fit, values between 0.08 and 0.10 represent a mediocre fit and values greater than 0.10 are not acceptable (Browne and Cudeck, 1993). The SRMR is the square root of the averaged squared residuals (i.e., the differences between the observed and predicted covariances). SRMR values < 0.10 are acceptable; however, values lower than 0.05 represent a good fit (Schermelleh-Engel et al., 2003). The CFI and the NNFI are incremental fit indices and measure the proportionate improvement in fit by comparing a target model with a baseline model (Hu and Bentler, 1999). The CFI compares the covariance matrix predicted by the model with the observed covariance matrix and compares the null model with the observed covariance matrix. The NNFI reflects the proportion by which the researcher's model improves the fit compared to the null model simultaneously controlling for the degrees of freedom. CFI and NNFI values greater than 0.97 are indicative of a good fit, whereas values greater than 0.95 may be interpreted as an acceptable fit (Schermelleh-Engel et al., 2003). The AIC statistic (= *X*^2^ − 2 *df*), which is a descriptive measure used to discriminate between competing models, was employed to compare the models. The models with the lowest AICs were considered to be the best.

**Figure 1:**
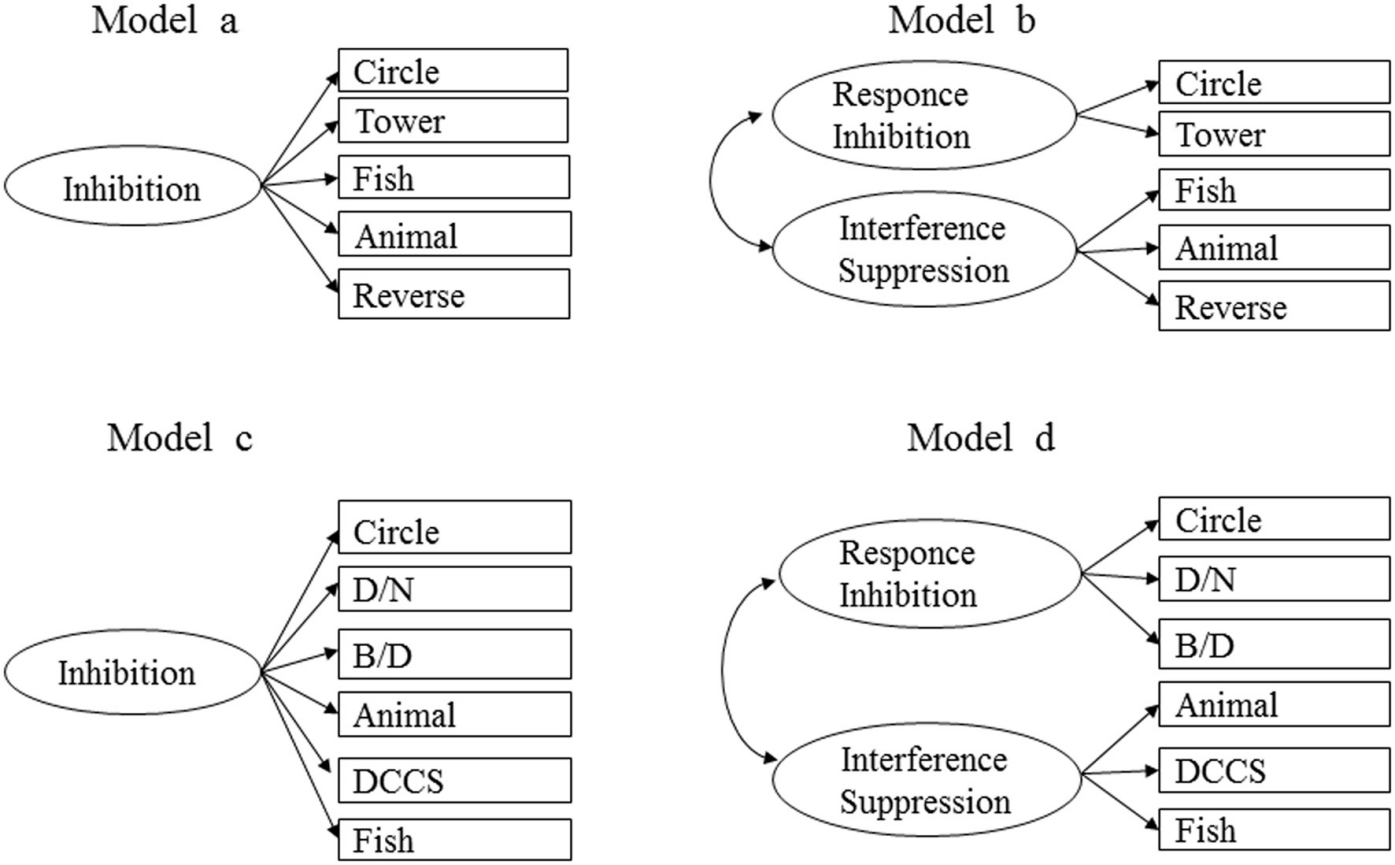
**Alternative CFA models of inhibition in toddlers**.

Considering data separately from both age groups, two different theoretical models were performed: an inhibition unitary model and a two-factor model, in which *response inhibition* and *interference suppression* were distinguished. Figure 1 schematically shows these comparative models. The fit indices for these models are summarized in Table 3.

**Table 3:** **Goodness of fit indices**.

**Group**	**Model**		**gdl**	***X*^2^**	***p***	**NNFI**	**CFI**	**RMSEA**	**SRMR**	**AIC**
24–32 months	**Model a**	**One Factor**	5	5.100	0.40	0.985	0.993	0.020	0.062	−4.900
36–48 months	Model c	One Factor	9	11.270	0.26	0.898	0.939	0.061	0.066	−6.730
	**Model d**	**Two Factors**	8	8.786	0.36	0.960	0.979	0.038	0.058	−7.214

*The endorsed models are indicated in bold*.

*RMSEA, root mean square error of approximation; SRMR, standardized root mean squared residual; CFI, comparative fit index; NNFI, non-normed fit index; AIC, akaike information criterion. The fit indices of Model b are not presented because the model did not converge*.

For the 24–32 month sample, the unitary model was the only acceptable solution. The two-factor model results showed that the value of the correlation between the two dimensions was 1; thus, it was not possible to run a model in which the two dimensions are distinguished.

The unitary model showed no significant *X*^2^ (*X*^2^ = 5.1, *p* = 0.40) and acceptable to good fit indices. Specifically, the NNFI, the CFI and the RMSEA values indicated good fits, and the SRMR showed an acceptable fit.

In contrast, in the oldest sample, the two-factor model fits the data better than the more parsimonious single-factor model. Although the estimate correlation between factors is high (*r* = 0.71; the 95% confidence interval for the correlation was [0.50, 0.84]), the two-factor model allows a better explanation of the observed data. As presented in Table 3, *X*^2^ was not significant in either solution (Model c, *X*^2^ = 11.27, *p* = 0.26; Model d, *X*^2^ = 8.79, *p* = 0.36). Nevertheless, the indices showed the best fit for the two-factor solution: the SRMR was acceptable in both models, however, in the two-factor model, the CFI, the NNFI and the RMSEA indicated good fits, whereas the same indices did not report acceptable values for the unitary model. Finally, the lowest AIC occurred for the two-factor model; thus, it showed the best fit.

**Figure 2:**
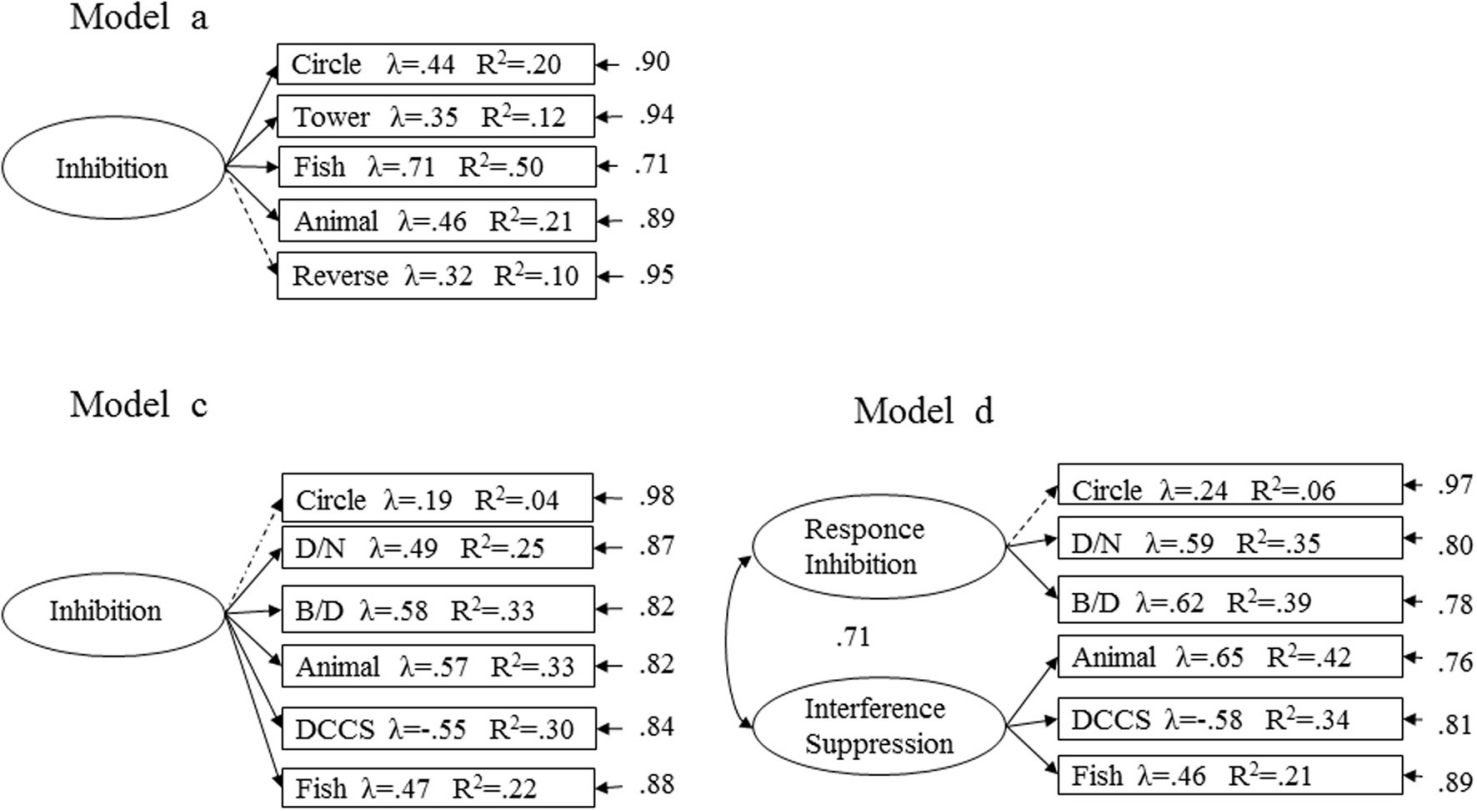
**Standardized factor loadings, R^2^ values and error terms are shown for unitary and two factor models for both age groups: model a for youngest children and model c and model d for oldest children**. The standardized factor loadings and the R^2^ values are reported into the box for each observed variable. The error terms are shown near the observed variables at the end of the smaller, single-headed arrows. No significant factor loadings are shown with dotted arrows. The correlation between the factors for model d is that next to the curved, double-headed arrow. Note: Circle, circle drawing task; Tower, tower building; Fish, fish task; Animal, animal house; Reverse, reverse categorization; Bear, bear and dragon; DCCS, dimensional change card sort; D/N, day/night stroop.

As reported in Figure 2, in both age ranges, the models identified significantly predicted all observed variables (*t* values >2) with the exception of the *Reverse Categorization* task in the 24–32 month sample and the *Circle Drawing Task* in the 36–48 month sample. The proportion of variability explained by the tasks varied from 0.12 to 0.50 in the youngest children and from 0.21 to 0.42 in the oldest children.

## Discussion

The aim of the current study was to examine the nature of inhibitory processes in early childhood. Although several authors have suggested a multifaceted nature of inhibition (Nigg, 2000; Friedman and Miyake, 2004; Clark et al., 2013; Diamond, 2013), an empirical investigation of the latent organization of inhibitory processes in early childhood was missing. The present investigation was an initial attempt to empirically assess the fit of two alternative models, which describe the latent structure of inhibition during the period from toddlerhood to preschool, a key transition point in children's development during which substantial gains occur in inhibitory task performance (Kochanska et al., 1997; Diamond, 2002; Jones et al., 2003; Carlson, 2005; Garon et al., 2008).

Though the growth of inhibitory control during childhood has been largely documented, especially in toddlerhood and preschool years (Diamond, 2002, for a review), no confirmatory analysis had previously been conducted to investigate the latent structure of the cognitive processes involved in inhibition.

In the present study, two samples of children (ranging in age from 24 to 32 months and from 36 to 48 months) from various socio-demographic backgrounds were assessed using age-appropriate inhibitory tasks involving different response demands. According to the literature, we considered two different models of inhibition development. First, we examined a unitary factor model, based on earlier studies that indicated a single undifferentiated executive control factor as the most appropriate for describing the executive latent structure in preschoolers (Wiebe et al., 2008, 2011; Hughes et al., 2010). Second, we examined a two-factor model, in which *response inhibition* was differentiated from *interference suppression*. The first component refers to the ability to control impulsive behavior and to prevent prepotent motor or verbal responses, whereas the second component involves more complex processes, such as WM, and comprises the suppression of interfering information. The separability of *response inhibition* and *interference suppression* has been described in older children (Bunge et al., 2002; Martin-Rhee and Bialystok, 2008).

In the 24–32 month sample, the simplest model with a single inhibition component was supported over the other model; the bi-factorial model, in which *response inhibition* and *interference suppression* were identified, was excluded because it was not acceptable due to the high correlation between the two latent factors. The unitary inhibition factor structure was chosen based on its relative and absolute model fits.

Parallel analyses were conducted for the 36–48 month sample. At this age level, the goodness-of-fit results indicated that a two-factor model provided the best fit to the data, with *response inhibition* as a separate dimension from an *interference suppression* factor.

The results suggest that inhibitory processes are not yet differentiated before 36 months of age, after which a distinction between different inhibitory dimensions emerges. We hypothesize a sequential development of inhibitory processes (see also Welsh et al., 1991; Espy, 1997; Espy et al., 2001; Senn et al., 2004): at an early age, the inhibition task performance primarily involves the ability to inhibit an impulsive or a dominant response (*response inhibition*); at a later stage, children develop a more cognitive inhibition that involves the suppression of interfering information or prepotent mental representations (*interference suppression*).

Tasks were selected to maximize the difference between *response inhibition* and *interference suppression*. As Miller et al. (2012) suggested, the task selection and the choice of performance indicators may influence the findings; consequently, both factors must be selected to clearly separate the different cognitive processes that must be assessed.

*Response inhibition* was evaluated in toddlers using the *Circle Drawing Task* and the *Tower Building* task, which require the ability to suppress prepotent but inappropriate responses. Similarly, in the *Bear/Dragon* task, the child needs to selectively suppress commanded actions. In particular, he/she must choose between two conflicting response types (performing or suppressing an action) based on a rule; however, the child must respond to a single stimulus which is clearly indicated by the experimenter. In the *Day/Night Stroop* task, the child must also suppress the tendency to produce a dominant response in relation to a target. Both tasks are thought to require inhibitory control and WM in remembering the rules. However, memory demands do not significantly influence the performance. With regards to the *Day/Night Stroop* task, Gerstadt et al. (1994) demonstrated that if children are asked to associate the labels “day” and “night” to two abstract designs, even preschoolers succeed. This condition still requires remembering two rules, but it does not require inhibiting the tendency to say what the stimuli really represent (Diamond et al., 2002). In the *Bear/Dragon* task, Jones et al. (2003) found that children between 36 and 48 months of age performed accurately on the activation trials, with the percent of correct responses at all ages ranging from 90 to 94 percent; in contrast, the accuracy in the inhibition trials increased with age, suggesting that the main difficulty in this task is suppressing a prepotent response. Therefore, these tasks all have in common the request to suppress a response that is solicited by the stimulus; the go responses become prepotent because they are habitual (Simpson and Riggs, 2006). Reck and Hund (2011) found that the *Bear/Dragon* task and the *Day/Night Stroop* task loaded on the same factor in a sample of preschool children aged 3–6 years, which suggests that the two tasks assessed similar cognitive processes.

*Interference suppression* was evaluated using the *Fish* and the *Animal House* tasks in both toddlers and preschoolers, the *Reverse Categorization* task in toddlers, and the *DCCS* in preschoolers. These tasks all require some level of response inhibition, similar to the previous tasks; however, they also require an individual to filter out incongruent information within the stimuli because children must respond to stimuli that contain both relevant and distracting information.

In case of the *Fish Task*, children need to control the impulse to touch the stimulus before they have observed the fish's direction (“*I mustn't touch the fish's food immediately but I have to observe the fish's direction before*”) (*response inhibition*); however, they also need to manage the visual and attentional interferences to solve the task. In particular, as suggested by Martin-Rhee and Bialystok (2008), this task requires the child to focus on one feature of the stimulus (the target fish direction) and ignore the other (the flankers' direction). This characteristic is present in all of the tasks that were chosen to assess *interference suppression*. The *Animal House* task requires children to control their impulsive behavior of putting all the stickers in the colored house without following any rules. Nevertheless, each time, they also need to select the right piece of information that is necessary to accomplish the task; for example, to correctly place the duck, the child must select the blue house and ignore the houses with other colors.

The *Reverse Categorization* task, which was used in the 24–32 month sample, is a sorting task that is very similar to the *DCCS* task, which was administered to children aged 36–48 months. Both tasks require children to classify objects or cards by considering their different features, the blocks' size (big or small) in the case of the *Reverse Categorization* and the color (red or blue) or the shape (rabbit or boat) in the case of the *DCCS*. Toddlers often fail to classify the blocks in the *Reverse Categorization* task in the post-switch phase (“*Now I've to put the big block in the small box*”) because they cannot inhibit the rule previously learned (“*I've to put the big block in the big box*”). Similarly, preschool children have difficulty switching from sorting by color to sorting by shape on the *DCCS* task because they have difficulty in inhibiting the old way of thinking about the objects. Children in the first year of preschool may remain stuck in thinking about objects according to the objects' initially relevant attribute (Diamond et al., 2005). The *DCCS* task requires high demands on the control of attention: children must inhibit their attention to a dimension that was previously valid to attend to a different aspect of the same stimulus.

The changes that occur in the nature of inhibitory processes from toddlerhood to early childhood may be due to both quantitative and qualitative changes in cognitive processing. For example, the *Fish* and *Animal House* tasks were explained by the same inhibitory dimension as the *Circle Drawing Task* in the 24–32 month sample. In contrast, in the 36–48 month sample, both tasks (i.e., the *Fish Task* and *Animal House* tasks) converged in the *interference suppression* factor, which suggests that at this age level, a child's performance is influenced by the ability to filter out irrelevant information, and not only by the ability to suppress a habitual response. Indeed, a specific task may not measure the same ability across different ages (Clark et al., 2013). For example, the *Tower of London* task (ToL), which is traditionally employed to assess planning in adults, proved to measure inhibitory control in young children (Bull et al., 2004).

As regards the reasons of the change in the organization of inhibitory processes across the two age-levels, this could be a result of maturational processes, as well as a consequence of the educational experiences of the children. While the 24–32 month sample was recruited from day-care centers, the children in the 36–48 month sample were attending the first year of preschool. In Italy, attendance at preschool is commonly accepted as the first essential stage of the educational system, and over 95% of children between 3 and 5 years of age attend a pre-primary school. Supporting school readiness, the preschool curriculum emphasizes activities that enhance creativity skills, social attitudes, autonomy and the learning process. The transition to preschool provides children with an opportunity to develop cognitive abilities and to improve self-regulation and executive function skills by increasing the children's participation in more structured activities that require more attentional control (Diamond and Lee, 2011; Hughes and Ensor, 2011).

However, the current results should be considered in the context of the study limitations.

First, because of the more limited behavioral repertoire of toddlers compared to preschool children, it was impossible to use exactly the same tasks in both age ranges. The tasks used in the 24–32 month sample are necessarily simpler, though they have similar inhibitory demands as the tasks used to assess the 36- to 48-month-old children. As indicated previously, the tasks used to assess *response inhibition* at both age levels comprised univalent stimuli associated with a prepotent response that must be overruled. While the tasks used to assess *interference suppression* comprised stimuli with different features, each was associated with a different response; thus, attention must be selectively focused on the relevant cue. Second, though the models tested were simple, the sample size at each age level was limited, suggesting that further evidence is needed to confirm our findings.

In conclusion, to the best of our knowledge, this is the first study to investigate the latent structure of inhibition at an early age. Because inhibition development is central in several theories of cognitive development (Dempster, 1992; Tipper, 1992; Harnishfeger and Bjorklund, 1993; Diamond and Taylor, 1996), the study of the nature of inhibitory processes from early childhood represents a significant area of research. Empirical evidence shows that children with typical development increase their performance in inhibition tasks from toddlerhood to the preschool period (Diamond, 2002; Carlson, 2005); at the same time, research has emphasized that a deficit in the development of inhibitory processes is associated with several psychopathological diseases, such as autism spectrum disorders (Ozonoff et al., 1991; Robinson et al., 2009) and attention deficit hyperactivity disorder (Barkley, 1997; Ozonoff and Jensen, 1999; Schachar et al., 2000). Finding an initial differentiation of inhibitory processes may be promising in understanding the development of inhibition in both typical and atypical developmental trajectories.

### Conflict of interest statement

The authors declare that the research was conducted in the absence of any commercial or financial relationships that could be construed as a potential conflict of interest.

## Footnotes

^1^EQS (6.1) [Computer software]. http://www.mvsoft.com/products.htm

## References

[B1] Bachorowski J. A. Newman J. P. (1985). Impulsivity in adults: motor inhibition and time-interval estimation. Pers. Individ. Dif. 6, 133–136 10.1016/0191-8869(85)90041-8

[B2] Barkley R. A. (1997). ADHD and the Nature of Self-control. New York, NY: Guilford Press

[B3] Bentler P. M. (2006). EQS 6 Structural Equations Program Manual. Encino, CA: Multivariate Software

[B4] Best J. R. Miller P. H. (2010). A developmental perspective on executive function. Child Dev. 81, 1641–1660 10.1111/j.1467-8624.2010.01499.x 21077853 PMC3058827

[B6] Blasi G. Goldberg T. E. Weickert T. Das S. Kohn P. Zoltick B. (2006). Brain regions underlying response inhibition and interference monitoring and suppression. Eur. J. Neurosci. 23, 1658–1664 10.1111/j.1460-9568.2006.04680.x 16553630

[B7] Browne M. W. Cudeck R. (1993). Alternative ways of assessing model fit, in Testing Structural Equation Models, eds Bollen K. A. Long J. S. (Beverly Hills, CA: Sage), 136–162

[B7a] Bull R. Espy K. A. Senn T. E. (2004). A comparison of performance on the Towers of London and Hanoi in young children. J. Child Psychol. Psychiatry 4, 743–754 10.1111/j.1469-7610.2004.00268.x 15056306

[B8] Bunge S. A. Dudukovic N. M. Thomason M. E. Vaidya C. J. Gabrieli J. D. E. (2002). Immature frontal lobe contributions to cognitive control in children: evidence from fMRI. Neuron 33, 301–311 10.1016/S0896-6273(01)00583-9 11804576 PMC4535916

[B10] Carlson S. M. (2005). Developmentally sensitive measures of executive function in preschool children. Dev. Neuropsychol. 28, 595–616 10.1207/s15326942dn2802_3 16144429

[B9] Carlson S. Mandell D. Williams L. (2004). Executive function and theory of mind: stability and prediction from ages 2 to 3. Dev. Psychol. 40, 1105–1122 10.1037/00121649.40.6.1105 15535760

[B12] Carlson S. M. Moses L. J. Breton C. (2002). How specific is the relation between executive function and theory of mind? Contributions of inhibitory control and working memory. Inf. Child Dev. 11, 73–92 10.1002/icd.298

[B13] Clark C. A. C. Sheffield T. D. Chevalier N. Nelson J. M. Wiebe S. A. Espy K. A. (2013). Charting early trajectories of executive control with the shape school. Dev. Psychol. 49, 1481–1493 10.1037/a0030578 23106846 PMC10860163

[B14] Dempster F. N. (1992). The rise and fall of the inhibitory mechanism: toward a unified theory of cognitive development and aging. Dev. Rev. 12, 45–75 10.1016/0273-2297(92)90003-K

[B16] Diamond A. (2002). Normal development of prefrontal cortex from birth to young adulthood: cognitive functions, anatomy, and biochemistry, in Principles of Frontal Lobe Function, eds Stuss D. T. Knight R. T. (New York, NY: Oxford University Press), 466–503 10.1093/acprof:oso/9780195134971.003.0029

[B17] Diamond A. (2013). Executive functions. Annu. Rev. Psychol. 64, 135–168 10.1146/annurev-psych-113011-143750 23020641 PMC4084861

[B18] Diamond A. Carlson S. M. Beck D. M. (2005). Preschool children's performance in task switching on the dimensional change card sort task: separating the dimensions aids the ability to switch. Dev. Neuropsychol. 28, 689–729 10.1207/s15326942dn2802_7 16144433 PMC1474810

[B20] Diamond A. Kirkham N. Amso D. (2002). Conditions under which young children can hold two rules in mind and inhibit a prepotent response. Dev. Psychol. 38, 352–362 10.1037/a0012593 12005379

[B21] Diamond A. Lee K. (2011). Interventions shown to aid executive function development in children 4-12 years old. Science 333, 959–964 10.1126/science.1204529 21852486 PMC3159917

[B22] Diamond A. Taylor C. (1996). Development of an aspect of executive control: development of the abilities to remember what I said and to “Do as I say, not as I do.” Dev. Psychobiol. 29, 315–334 10.1002/(SICI)1098-2302(199605)29 8732806

[B24] Eriksen B. A. Eriksen C. W. (1974). Effects of noise letters upon the identification of a target letter in a nonsearch task. Percept. Psychophys. 16, 143–149 10.3758/BF03203267

[B25] Espy K. A. (1997). The shape school: assessing executive function in preschool children. Dev. Neuropsychol. 13, 495–499 10.1080/87565649709540690

[B26] Espy K. A. Kaufmann P. M. Glisky M. L. McDiarmid M. D. (2001). New procedures to assess executive functions in preschool children. Clin. Neuropsychol. 15, 46–58 10.1076/clin.15.1.46.1908 11778578

[B27] Friedman N. P. Miyake A. (2004). The relations among inhibition and interference control functions: a latent-variable analysis. J. Exp. Psychol. Gen. 133, 101–135 10.1037/0096-3445.133.1.101 14979754

[B29] Fuhs M. W. Day J. D. (2011). Verbal ability and executive functioning development in preschoolers at Head Start. Dev. Psychol. 47, 404–416 10.1037/a0021065 21142363

[B30] Garon N. Bryson S. Smith I. (2008). Executive function in preschoolers: a review using an integrative framework. Psychol. Bull. 134, 31–60 10.1037/0033-2909.134.1.31 18193994

[B31] Gerstadt C. L. Hong Y. J. Diamond A. (1994). The relationship between cognition and action: performance of children 3 ½ -7 years old on a Stroop-like day-night test. Cognition 53, 129–153 10.1016/0010-0277(94)90068-X 7805351

[B32] Harnishfeger K. K. Bjorklund D. F. (1993). The ontogeny of inhibition mechanism: a renewed approach to cognitive development, in Emerging Themes in Cognitive Development, eds Howe M. L. Pansak R. (New York, NY: Springer-Verlag), 28–49

[B34] Hu L. Bentler P. M. (1999). Cutoff criteria for fit indexes in covariance structure analysis: coventional criteria versus new alternatives. Struct. Eq. Model. 6, 1–55 10.1080/10705519909540118

[B35] Hughes C. Ensor R. (2011). Individual differences in growth in Executive Function across the transition to school predict externalizing and internalizing behaviors and children's self-perceived academic success at age 6. J. Exp. Child Psychol. 108, 663–676 10.1016/j.jecp.2010.06.005 20673580

[B36] Hughes C. Ensor R. Wilson A. Graham A. (2010). Tracking executive function across the transition to school: a latent variable approach. Dev. Neuropsychol. 35, 20–36 10.1080/87565640903325691 20390590

[B39] Jones L. B. Rothbart M. K. Posner M. I. (2003). Development of executive attention in preschool children. Dev. Sci. 6, 498–504 10.1111/1467-7687.00307

[B41] Kochanska G. Murray K. Coy C. K. (1997). Inhibitory control as a contributor to conscience in childhood: from toddler to early school age. Child Dev. 68, 263–277 10.2307/1131849 9180001

[B42] Kochanska G. Murray K. Jacques T. Y. Koenig A. L. Vandegeest K. A. (1996). Inhibitory control in young children and its role in emerging internalization. Child Dev. 67, 490–507 10.1111/j.1467-8624.1996.tb01747.x 8625724

[B43a] Lehto J. E. Juujärvi P. Kooistra L. Pulkkinen L. (2003). Dimensions of executive functioning: evidence from children. British J. Dev. Psychol. 21, 59–80 10.1348/026151003321164627

[B44] Martin-Rhee M. M. Bialystok E. (2008). The development of two types of inhibitory control in monolingual and bilingual children. Biling Lang. Cogn. 11, 81–93 10.1017/S1366728907003227

[B45] Miller M. R. Giesbrecht G. F. Müller U. McInerney R. J. Kerns K. A. (2012). A latent variable approach to determining the structure of executive function in preschool children. J. Cogn. Dev. 13, 395–423 10.1080/15248372.2011.585478

[B45a] Miyake A. Friedman N. P. Emerson M. J. Witzki A. H. Howerter A. Wager T. D. (2000). The unity and diversity of executive functions and their contributions to complex “frontal lobe” tasks: a latent variable analysis. Cogn. Psychol. 41, 49–100 10.1006/cogp.1999.0734 10945922

[B47] Nigg J. T. (2000). On inhibition/disinhibition in developmental psychopathology: views from cognitive and personality psychology and a working inhibition taxonomy. Psychol. Bull. 126, 220–246 10.1037/0033-2909.126.2.220 10748641

[B48] Ozonoff S. Jensen J. (1999). Specific executive function profiles in three neurodevelopmental disorders. J. Autism Dev. Disord. 29, 171–177 10.1023/A:1023052913110 10382139

[B49] Ozonoff S. Pennington B. F. Rogers S. J. (1991). Executive function deficits in high-functioning autistic individuals: relationship to theory of mind. J. Child Psychol. Psychiatry 32, 1081–1105 10.1111/j.1469-7610.1991.tb00351.x 1787138

[B50] Reck S. G. Hund A. M. (2011). Sustained attention and age predict inhibitory control during early childhood. J. Exp. Child Psychol. 108, 504–512 10.1016/j.jecp.2010.07.010 20801457

[B51] Reed M. Pien D. L. Rothbart M. K. (1984). Inhibitory self-control in preschool children. Merrill. Palmer. Q. 30, 131–147

[B52] Robinson S. Goddard L. Dritschel B. Wisley M. Howlin P. (2009). Executive function in children with autism spectrum disorders. Brain Cogn. 71, 362–368 10.1016/j.bandc.2009.06.007 19628325

[B53] Rueda M. R. Fan J. McCandliss B. D. Halparin J. D. Gruber D. B. Lercari L. P. (2004). Development of attentional networks in childhood. Neuropsychologia 42, 1029–1040 10.1016/j.neuropsychologia.2003.12.012 15093142

[B54] Rueda M. R. Posner M. I. Rothbart M. K. (2005). The development of executive attention: contributions to the emergence of self-regulation. Dev. Neuropsychol. 28, 573–594 10.1207/s15326942dn2802_2 16144428

[B55] Schachar R. Mota V. L. Logan G. D. Tannock R. Klim P. (2000). Confirmation of an inhibitory control deficit in attention-deficit/hyperactivity disorder. J. Abnorm. Child Psychol. 28, 227–235 10.1023/A:1005140103162 10885681

[B56] Schermelleh-Engel K. Moosbrugger H. Müller H. (2003). Evaluating the fit of structural equation models: tests of significance and descriptive goodness-of-ft measures. Meth. Psychol. Res. 8, 23–74 Available online at: http://www.mpr-online.de

[B57] Senn T. E. Espy K. A. Kaufmann P. M. (2004). Using path analysis to understand executive functions organization in preschool children. Dev. Neuropsychol. 26, 445–464 10.1207/s15326942dn2601_5 15276904

[B58] Simpson A. Riggs K. J. (2006). Conditions under which children experience inhibitory difficulty with a “button-press” go/no-go task. J. Exp. Child Psychol. 94, 18–26 10.1016/j.jecp.2005.10.003 16325846

[B60] Simpson, A, Riggs K. J Beck S. R Gorniak S. L Wu Y. Abbott D. (2012). Refining the understanding of inhibitory control: how response prepotency is created and overcome. Dev. Sci. 15, 62–73 10.1111/j.1467-7687.2011.01105.x 22251293 PMC3405835

[B61] Tipper S. P. (1992). Selections for actions: the role of inhibitory mechanisms. Curr. Dir. Psychol. Sci. 1, 105–112 10.1111/1467-8721.ep10768813

[B62] Usai M. C. Viterbori P. Traverso L. De Franchis V. (2014). Latent structure of executive function in 5-to and 6-year-old children: a longitudinal study. Eur. J. Dev. Psychol. 11, 447–462 10.1080/17405629.2013.840578

[B63] Viterbori P. Gandolfi E. Usai M. C. (2012). Executive skills and early language development. J. Appl. Psycholinguist. 3, 17–32

[B64] Wechsler D. (1973). Wechsler Preschool and Primary Scale of Intelligence—Italian Version. Firenze: Organizzazioni Speciali

[B65] Welsh M. C. Pennington B. F. Groisser D. B. (1991). A normative-developmental study of executive function: a window on prefrontal function in children. Dev. Neuropsychol. 7, 131–149 10.1080/87565649109540483

[B66] Wiebe S. Espy K. Charak D. (2008). Using confirmatory factor analysis to understand executive control in preschool children: I. Latent structure. Dev. Psychol. 44, 575–587 10.1037/0012-1649.44.2.575 18331145

[B67] Wiebe S. Sheffield T. Nelson J. M. Clark C. A. C. Chevalier N. Espy K. (2011). The structure of executive function in 3-year-olds. J. Exp. Child Psychol. 108, 436–452 10.1016/j.jecp.2010.08.008 20884004 PMC3033982

[B68] Willoughby M. T. Blair C. B. Wirth R. J. Greenberg M. (2010). The measurement of executive function at age 3 years: psychometric properties and criterion validity of a new battery of tasks. Psychol. Assess. 22, 306–317 10.1037/a0018708 20528058

[B69] Zelazo P. D. (2006). The dimensional change card sort (DCCS): a method of assessing executive function in children. Nat. Prot. 1, 297–301 10.1038/nprot.2006.46 17406248

